# Assessment of a Digital Platform for Routine Outcome Monitoring in Psychotherapy: Usability Study and Thematic Analysis

**DOI:** 10.2196/75885

**Published:** 2025-09-30

**Authors:** Mattia Vincenzo Olive, Antonino La Tona, Gianluca Lo Coco, Angelo Compare, Joseph Antony Cafazzo, Cristina Masella

**Affiliations:** 1 Department of Management, Economics and Industrial Engineering, Politecnico di Milano, Via Raffaele Lambruschini 4B, Milano, 20156, Italy, 39 3488676027; 2 Department of Human and Social Sciences, University of Bergamo, Bergamo, Italy; 3 Department of Psychology, Educational Science and Human Movement, University of Palermo, Palermo, Italy; 4 Healthcare Human Factors; Centre for Digital Therapeutics, University Health Network, Toronto, ON, Canada; 5 Institute of Health Policy, Management and Evaluation, University of Toronto, Toronto, ON, Canada

**Keywords:** digital mental health, psychotherapy, routine outcome monitoring, usability, digital tools

## Abstract

**Background:**

The integration of digital tools into psychotherapy has gained increasing attention, particularly for practices such as routine outcome monitoring (ROM), which involves the regular collection of patient-reported data to inform treatment decisions. However, despite the potential benefits, the adoption of digital platforms remains limited, partly due to usability concerns and workflow misalignment.

**Objective:**

This study aimed to assess the usability of a digital platform, Mindy, designed to support psychotherapists in implementing ROM and to explore broader challenges associated with the integration of digital tools into psychotherapeutic practice.

**Methods:**

This study adopted a qualitative, 2-stage approach. Sixteen psychotherapists participated in semistructured interviews, which included task-based usability testing and reflective discussions. Participants interacted with Mindy by performing typical clinical tasks, such as creating patient profiles, managing session data, and sending questionnaires. The first stage of analysis used a deductive thematic approach focused on predefined platform functionalities. The second stage followed an inductive methodology to identify broader themes related to the integration of digital tools in psychotherapy.

**Results:**

The usability assessment identified strengths in the platform’s appointment scheduling, questionnaire delivery, and dashboard functionalities, which were perceived as intuitive and supportive of ROM practices. However, limitations were reported in areas such as documentation flexibility, interoperability with other systems, and control over information sharing with patients. Broader thematic analysis revealed three main challenges: (1) the tension between standardized documentation and the need for narrative and implicit information; (2) difficulties in embedding digital platforms into existing therapeutic workflows, especially for clinicians less familiar with technology; and (3) concerns about confidentiality and the potential for misinterpretation when sharing therapeutic notes with patients.

**Conclusions:**

These findings underscore the importance of considering both technical and contextual dimensions when developing and implementing digital platforms in mental health care. Tailoring digital tools to the needs and practices of psychotherapists may improve adoption and ultimately enhance the quality of care.

## Introduction

Digital technologies, such as electronic health records (EHRs) and digital mental health platforms (DMHPs), have become increasingly relevant in psychotherapy [1-3]. These technologies offer several benefits, such as improved documentation and legibility, enhanced information sharing and communication among providers, timely information retrieval, and increased accountability [4]. However, their implementation also presents several challenges, including issues with standardization and codification, the time burden placed on clinicians, potential negative impacts on therapeutic relationships, usability and learning curve difficulties, and privacy concerns [4]. Certain practices and approaches in psychotherapy are notably enhanced by digital technologies, with routine outcome monitoring (ROM) being a prime example [5]. ROM has been studied and implemented under different names, including “progress monitoring” [6], “feedback-informed treatment” [7], and “outcome monitoring feedback” [8,9], “measurement-based care” [10], and “outcome feedback” [11]. ROM consists of the systematic collection and analysis of patient progress and outcome data throughout therapy sessions, using standardized self-report measures at regular intervals to provide valuable insights to optimize treatment strategies. The information derived from these measurements is typically presented in graphical form and provided to the therapist and possibly the patient to facilitate monitoring of the patient’s improvement trajectory over time. Historically, implementing ROM has been hindered by challenges such as the continuous monitoring of patients and the timely delivery of feedback to therapists [5]. The adoption of digital solutions, however, has been shown to significantly mitigate these obstacles, facilitating a more efficient process [5]. Before these innovations, the collection of patient data was predominantly based on paper-based practices, which were often incomplete, lacked comprehensiveness, and were prone to memory bias [5,12]. The advent of digital technologies has enabled real-time collection of more detailed and objective patient data, allowing therapists to assess information both before and after the visit and thereby reducing reliance on patient memory [13].

The American Psychological Association (APA) has long advocated the use of ROM and feedback practices in psychological therapy [14]. A recent advisory committee appointed by APA governance further recommended the creation of comprehensive guidelines regarding the integration of outcome and process monitoring systems into therapeutic practice. These guidelines state that therapists should undertake regular evaluations of the treatment process and outcomes, integrating this evidence-based practice into clinical practice [12].

Despite substantial scientific evidence supporting the clinical benefits of ROM, its adoption remains limited in certain contexts. This hesitation can be attributed to several factors, such as the lack of tailored validation studies, insufficient technological infrastructure, and cultural resistance within clinical practice. Moreover, there is a broader challenge of translating evidence-based approaches into actionable practices in health care systems that may be underresourced or overly reliant on traditional methods [15].

This research is part of the broader OutProFeed project, which is conducting the first randomized controlled trial in Italy focused on ROM.

Within the framework of this larger initiative, the paper addresses 2 specific objectives. The first is to assess the usability of Mindy, an integrated EHR-DMHP platform, designed to support ROM implementation. This usability assessment aims to evaluate how effectively the platform aligns with clinicians’ workflows [16,17].

The second objective stems from a broader reflection on the current state of research on digital platforms in mental health. While the effectiveness of these tools for data collection and patient monitoring is widely addressed in the literature [18], often focusing on their technical functionalities, advantages, disadvantages, and impacts on clinical outcomes [18], there is limited evidence on how psychotherapists integrate such platforms into their daily routines [2,19,20]. As a result, adopting a holistic and integrated approach to the design and implementation of digital tools in mental health appears increasingly important. Through the specific case of Mindy’s use of ROM, this research aims to contribute to this underexplored area by analyzing the challenges involved in integrating digital platforms into psychotherapeutic practice.

## Methods

### Overview

This study used a single dataset but followed a 2-pronged data analysis approach to address distinct objectives. The research began by assessing the usability [16,17] of the Mindy digital platform, focusing on its features and collecting feedback from psychotherapists. A deductive thematic analysis [21-23] was applied to organize the data into predefined categories aligned with the platform’s features, such as appointment scheduling, patient profile management, and session data entry. The findings were synthesized into actionable recommendations for improving the platform, which were communicated to Mindy’s technical and development teams during 2 feedback stages: after a preliminary usability testing phase and following the main evaluation.

However, during this usability analysis, recurring patterns emerged that extended beyond platform features, pointing to broader challenges in integrating digital tools into psychotherapeutic workflows. Recognizing the significance of these themes, the research team conducted a second round of data analysis using an inductive approach [21,23,24]. This phase revisited the data without predefined categories, identifying emerging themes, grouping them into broader concepts, and ultimately into aggregate dimensions. This 2-stage analysis allowed the study to address both the usability of the platform and the deeper, systemic challenges faced by psychotherapists when incorporating digital tools into their daily practice.

The following sections provide a detailed description of the Mindy platform’s features, outline the data collection process, and explain the 2-stage data analysis approach.

### Key Features of the Platform Under Assessment

Mindy is the digital platform under assessment, and its functionalities include a calendar for scheduling appointments, a document management system for tasks such as handling informed consent forms, session reports, and patient management. It also features an integrated system for administering questionnaires, which provides automatic and standardized scoring of copyright-free measures. In addition, the platform includes a dashboard for monitoring patient progress and the therapeutic alliance, based on patient responses to predefined, validated questionnaires. An overview of the features is provided in Table 1, while screenshots of the main interface for these features are available in Multimedia Appendix 1.

**Table 1.** Main features of the “Mindy” platform.

Feature	Description
Agenda and appointment booking	The platform provides therapists with access to a calendar in monthly or weekly views, enabling the scheduling of patient appointments in both in-person and remote modes. Upon booking, an email reminder is automatically sent to both the patient and therapist. For remote appointments, the system also sends the patient a link to the virtual meeting room.
Patient profile creation	Therapists can use the platform to register new patients, collect their personal information, and create a dedicated folder for each individual. Following registration, an informed consent form is automatically emailed to the patient, who must download, sign, and return it electronically or in paper format.
Management of session data with the patient	The platform includes a section for recording details of patient visits, with fields available for annotations and attachments (free-text notes); mandatory fields (eg, current symptoms, examination findings, and preliminary diagnosis) that must be completed to finalize the visit and generate a report; detailed data (optional fields for lifestyle recommendations, observations, or care plans); and questionnaires (predefined forms that can be automatically delivered to patients, with timing specified by the therapist and responses viewable in a dedicated section).
Report delivery to patients	The platform enables therapists to generate and send reports to patients, primarily containing information recorded in the mandatory fields
Dashboard to monitor patient progress and the therapeutic alliance	The platform includes a dashboard where therapists can track patient progress and assess the therapeutic relationship. For instance, therapists can send predefined questionnaires to patients to monitor their condition over time. The results are automatically processed by the platform and presented graphically for review.
Online session system	The platform facilitates online therapy sessions by allowing therapists to initiate video calls directly from the system. A link to the virtual meeting is automatically sent to the patient before the session begins.

### Data Collection Process

The data collection process involved semistructured interviews with psychotherapists (refer to Multimedia Appendix 1 for the interview outline), conducted remotely via Microsoft Teams to ensure flexibility and allow direct observation of participants interacting with the platform.

Each session lasted 30‐45 minutes and followed a 2-part structure. In the first part, participants were asked to complete tasks simulating typical psychotherapeutic activities, such as creating a patient profile, scheduling an appointment, filling out a session report, and sending a questionnaire. These tasks were selected to range from relatively straightforward but essential for basic platform use to more complex tasks critical for implementing ROM through the platform. This progression was designed to reflect both the importance of these activities in psychotherapeutic workflows and their potential to highlight areas where users might encounter challenges.

During these tasks, the thinking-aloud approach [25-27] was used, encouraging participants to verbalize their thoughts while navigating the platform. This approach provided insights into how participants interacted with the system and identified any usability challenges. Interviewers intervened only when participants struggled to articulate their thoughts, using prompts such as, “What are you thinking?” or “What are you trying to do?” In the second part, participants stopped screen sharing to respond to questions exploring their experiences with the platform and its potential integration into their clinical workflow. These questions sought feedback on specific features and how the platform could fit into daily practice.

To ensure familiarity, participants were given 3 days before the interview to explore the platform independently, performing activities such as creating patient profiles or scheduling appointments. Before each session, interviewers clarified that the study aimed to evaluate the platform, not the digital skills of the participants, fostering a noncritical and supportive environment. The choice of a qualitative methodology to assess usability was driven by its capacity to provide detailed insights regarding the usability of Mindy, emphasizing the reasons behind observed behaviors and the contextual factors shaping them [26,28]. Unlike quantitative approaches, which prioritize measurable metrics such as task completion times or error rates [16,17,29], the qualitative method allows for a richer analysis of user interactions [28]. This approach was considered most appropriate for the objectives of the study, as it facilitated the identification of usability issues while simultaneously offering a deeper understanding of how the platform could be integrated into psychotherapists’ daily workflows.

All interviews were recorded with consent to capture verbal and nonverbal data for later analysis. Informed written consent was obtained from all participants before their inclusion in the study.

### Sample

Among those who participated in the broader OutProFeed project, 16 psychotherapists were recruited for this study using a convenience sampling approach. Recruitment and interviews were conducted from June 15, 2024, to July 20, 2024. No maximum number of interviews was predefined, as the focus was on reaching data saturation [30], ensuring that additional interviews would not yield new insights. However, a minimum number of 15 participants was established based on Food and Drug Administration (FDA) guidelines [31], which emphasize the importance of collecting sufficient data to identify usability issues and gather comprehensive feedback.

A preusability testing phase was conducted with 3 therapists to pilot the initial methodology, which involved only semistructured interviews without direct interaction with the platform. This phase highlighted the limitations of the initial approach, as the lack of hands-on interaction made it difficult to obtain detailed and actionable feedback. Consequently, the methodology was revised to include a user testing phase using the thinking-aloud method, enabling therapists to interact directly with the platform while verbalizing their thoughts.

The psychotherapists who participated in the study had varying levels of professional experience, with the majority (n=8) reporting 1‐4 years of practice, followed by those with 5‐9 years (n=6), and a smaller group with over 10 years (n=1). The sample was predominantly female, with 15 of 16 participants (94%) identifying as women and only 1 of 16 (6%) as a man. In terms of specialization, most participants practiced cognitive behavioral therapy (n=8), while others specialized in transactional analysis (n=4) and integrated approaches (n=4). This distribution reflects a diverse sample in terms of experience and therapeutic orientation, providing a well-rounded perspective for the study.

### Two-Round Data Analysis

The data analysis followed a 2-stage approach. In the first stage, a deductive approach was used [21-23], guided by Braun and Clarke’s [32] thematic analysis methodology. This phase involved coding the data and organizing it into themes directly linked to the predefined features of the Mindy platform, such as appointment scheduling, patient profile creation, and session data management. This structured approach ensured that participants’ feedback was systematically categorized according to the platform’s functionalities, allowing for a targeted assessment of usability issues and areas for improvement.

**Figure 1.**
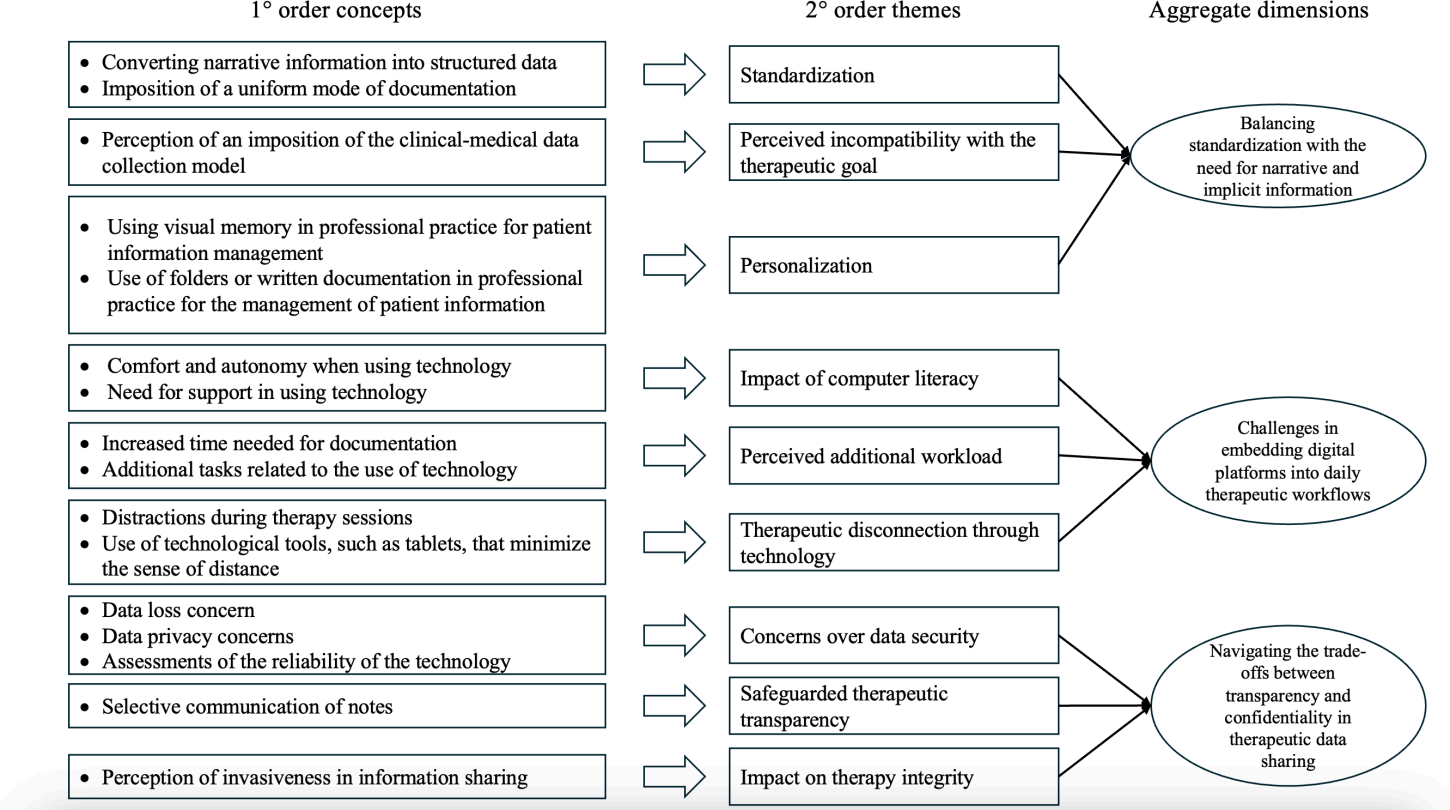
Data structure.

The second stage used an inductive approach [21,23,24], based on the Gioia methodology [33], to explore broader themes that emerged from the data. Open coding was used to identify first-order concepts, which were subsequently grouped into second-order themes and finally into aggregate dimensions. The data structure is provided in Figure 1.

### Ethical Considerations

This study received ethical approval from the Comitato per l'Integrità e l'etica della ricerca of the University of Bergamo (reference no 2024_04_08) during the meeting held on April 15, 2024 (minutes no 04/2024). The committee issued a favorable opinion without identifying significant critical issues regarding the study design or execution. All procedures were conducted in accordance with institutional and national ethical standards and adhered to the Declaration of Helsinki. Informed written consent was obtained from all participants before their inclusion in the study. Participants were provided comprehensive information regarding the study's nature, purpose, procedures, and data usage. The privacy and confidentiality of all participants were rigorously protected. Video interviews were conducted, and all recordings are securely stored in the institutional archives of Politecnico di Milano in compliance with European privacy regulations, General Data Protection Regulation (GDPR), and ethical committee requirements, with access restricted to authorized research personnel only. No financial or material compensation was offered or provided to the participants in this study.

## Results

### Overview

The results are presented and discussed in 2 parts. The first part focuses on the usability study, outlining the issues identified and linking them to specific functionalities of the Mindy platform. The second part explores broader themes that emerged from the data, highlighting the challenges of integrating digital platforms into psychotherapeutic practice.

### Results From the Usability Study

To organize the feedback collected, a summary table was created (Table 2), which groups usability evaluations by platform functionality. The rows of the table represent the various functionalities of the platform, while the columns are divided into 3 categories: positive evaluations, negative evaluations, and suggestions for improvement.

Each intersection between a functionality and a category includes detailed descriptions:

- “Positive evaluations” highlight aspects of the functionality that therapists found effective or beneficial.
- “Negative evaluations” describe elements identified as problematic or inadequate.
- “Suggestions for improvement” provide recommendations for enhancing the usability of the functionality.

**Table 2.** Results from usability assessment.

Functionality	Positive evaluation	Negative evaluation	Suggestions for improvement
Agenda and appointment booking	Intuitive and easy-to-navigate layout; automatic reminders sent 24 hours before appointments streamline scheduling	Limited flexibility: no integration with external calendars; restricts comprehensive appointment management across platforms	Consider integrating with external calendars (eg, Google Calendar) to provide a unified view of schedules; this feature would enhance organization and reduce scheduling conflicts
Patient profile creation	Comprehensive data collection aligns with therapists’ practices; quick and clear data entry ensures ease of use	Gender options are restricted to male and female; inability to attach identity document scans complicates work, especially for minors	Add an “Other” option for gender selection to accommodate diverse patient identities; enable attachment of scanned identity documents for compliance and better record-keeping
Automatic sending of the informed consent form	Easy-to-use feature that automatically sends forms after patient data is entered; customizable templates allow personalization for different therapists	Requires physical printing, signing, and scanning of consent forms, reducing digital efficiency	Enable patients to upload signed forms directly via a secure link; add support for digital signatures to allow seamless consent management; facilitate secure digital storage of signed forms to eliminate paper documentation
Annotations and attachments section	Flexible, unstructured format allows personalized and spontaneous note-taking; supports diverse documentation needs during or after sessions	Requires a laptop, which can distract therapists during sessions; incompatible with tablets or digital pens, limiting usability for many practitioners; online-only access risks data inaccessibility during connection issues	Enable uploading of scanned handwritten notes or photos; ensure compatibility with tablets and support digital pen input for flexible note-taking; introduce an offline mode or a synchronization feature to maintain access during outages
Mandatory fields and detailed data sections	Helps therapists remember to document important details during sessions	Requires frequent updates to rarely changing data (eg, medical history), leading to inefficiencies; overly medicalized structure does not align with all therapy needs	Make certain fields permanent for stable information, avoiding repeated updates; add a feature for incremental updates, enabling therapists to log changes only when necessary
Dashboard to monitor patient progress and therapeutic alliance	Graphical progress display is intuitive and provides a clear visual overview; it helps therapists quickly identify patient progress or regression	Graphs lack dates, limiting the ability to contextualize progress over time	Add dates to the horizontal axis of graphs, linking progress to specific sessions or events; this improvement would enhance trend analysis and treatment planning
Sending questionnaires	Fast and efficient process that allows results to be stored directly in the patient’s file; simplifies data collection and organization	No option to include custom questionnaires, reducing flexibility for therapists	Allow therapists to upload and manage custom questionnaires in a dedicated section; this would increase adaptability to specific patient needs
Sending reports to patients	Automatic report generation is quick and easy to use	Therapists cannot control what content is shared in the report; reports are sent automatically after each session, limiting flexibility in timing; lack of control can cause confusion if all details are not relevant for immediate discussion	Allow therapists to customize which information is shared in the report; make report sending optional, giving therapists control over the timing and content; this flexibility would improve communication and align report sharing with therapeutic goals

### Challenges of Integrating Digital Platforms Into Psychotherapeutic Practice

Three main themes emerged from the inductive analysis of the data, offering insights into the challenges of integrating a digital platform such as Mindy into psychotherapeutic practice: (1) balancing standardization with the need for narrative and implicit information; (2) embedding digital platforms into daily therapeutic workflows; and (3) navigating the trade-offs between transparency and confidentiality in therapeutic data sharing.

With respect to ROM practices, the feedback gathered on dashboards to monitor patient progress and questionnaire management (Table 1) was particularly relevant. These features are central in ROM practices enabled by digital systems, as they allow easy and intuitive management of clinical data on outcomes and therapeutic processes. These tools increase the therapist’s responsiveness [34], which in turn can impact patient change from one session to the next [35]. The literature has shown that ROM data, especially those managed by digital systems, can increase therapists’ awareness of how they have affected patients, improve their humility and empathy, increase patients’ understanding of themselves, and involve them more actively in treatment [36]—aspects that influence the quality of therapy [8]. The perceived importance and usefulness of ROM methods by therapists will influence their adoption [37]. Therefore, any implementation efforts should seek to improve awareness and understanding by providing data on the empirical basis and clinical efficacy of ROM as an evidence-based practice, which enables personalization of therapy, improves outcomes even in the most difficult cases, and reduces patient drop-out rates [34].

#### Balancing Standardization With the Need for Narrative and Implicit Information

The Mindy platform introduces a structured system for psychotherapeutic documentation that promotes uniformity and consistency. However, this shift has raised concerns about the loss of flexibility and personalization that are central to psychotherapeutic practice. Traditionally, therapists relied on free-text notes to capture nuanced details such as tone, body language, and contextual observations. In contrast, Mindy requires the use of structured fields, drop-down menus, and predefined scales, which some therapists find limiting. One therapist commented:

*Instead of having a window with options for the objective examination, it would be more effective to use an open-ended question. That way, after formulating a diagnostic hypothesis, I would have the opportunity to add arguments and details to support that initial diagnosis.*

The rigidity of standardized documentation risks oversimplifying therapeutic interactions and reducing the richness needed to fully understand a patient’s unique case. A therapist observed:

*This section, in my opinion, is completely useless unless one works exactly with DCA and decides to use this questionnaire. I, for example, would not use it.*

While some therapists appreciate the structured fields for reminding them to cover key aspects during sessions, others emphasize the importance of flexibility. One explained:

*Sometimes one leaves room for the flow, not the free association of the patient, and forgets to ask determined things.*

To address these concerns, introducing customizable templates and free-text options could help Mindy balance standardization with the flexibility therapists require. Such changes would allow therapists to retain the depth and personalization critical to effective documentation without compromising consistency.

#### Challenges in Embedding Digital Platforms Into Daily Therapeutic Workflows

Integrating Mindy into psychotherapists’ daily workflows presents challenges, particularly for those accustomed to paper-based methods. Many therapists perceive the transition as requiring significant effort and time, which they already find scarce. One therapist noted:

*Compared to what I need, it asks me for a major effort, so I don’t do it.*

Computer literacy also plays a role in adoption resistance, especially among experienced therapists less familiar with digital tools. Despite some willingness to adapt, as one therapist stated:

*It might be useful, but I would have to change my way of working a little bit, being very paper-based.*

Therapists also expressed concerns about how the use of digital tools during sessions might create a barrier between them and their patients. For instance, one therapist remarked:

*When I do the examination, I don’t keep the desk in front. I stand in the chairs in front of the patient, so I don’t have anything technological in front of me.*

Another added:

*Using the PC unhooks me too much. I tried it, but it creates too much of a boundary between me and the patient.*

Data security is another significant concern, as therapists are responsible for safeguarding highly sensitive information. Any perceived vulnerabilities in the platform’s security could deter adoption. Addressing these challenges requires a user-centered design approach, targeted training, and direct engagement with therapists to ensure that the platform aligns with their workflows and supports, rather than disrupts, their practice.

#### Navigating the Trade-Offs Between Transparency and Confidentiality in Therapeutic Data Sharing

Mindy’s secure environment for managing therapeutic data offers significant advantages, but sharing session notes with patients raises concerns about misinterpretation. Traditionally, therapists’ notes were intended for personal use, containing preliminary observations and reflections that could be misunderstood if taken out of context. One therapist explained:

*I never send notes to the patient unless they specifically ask me. Otherwise, they might misunderstand what I’m writing.*

Unmediated sharing of notes risks undermining the therapeutic alliance by introducing confusion or unnecessary anxiety. A reflective comment, for example, might be mistaken for a definitive diagnosis, potentially straining the trust between therapist and patient. Another therapist elaborated:

*Sometimes I write things that are hypotheses, ideas to explore further, and I wouldn’t want the patient to think those are fixed conclusions. It could create unnecessary worry.*

To mitigate these risks, Mindy should include customizable sharing options that allow therapists to control what information is shared with patients and when. This would preserve the confidentiality and trust essential to effective therapy while enabling transparency where appropriate.

## Discussion

### Overview

This study aimed to evaluate the usability of the Mindy platform and explore broader challenges in integrating digital tools into psychotherapeutic workflows. Usability has long been recognized as a critical factor in the adoption and effectiveness of digital health technologies [16,17]. By combining a usability evaluation with an exploration of systemic challenges, the study builds on existing research emphasizing the importance of human-centered design and contextual integration [37,38]. Platforms such as Mindy hold significant potential to enhance therapeutic practices, particularly in supporting ROM, but the integration of such tools often encounters barriers related to design, workflow alignment, and therapist adoption. Addressing these challenges is essential for maximizing the benefits of digital health technologies in psychotherapy.

### On the Usability of Digital Platforms in Psychotherapy

The usability evaluation of the Mindy platform identified a range of strengths and weaknesses in its design, offering valuable insights for broader considerations in developing and implementing DMHPs. Although this study focused on Mindy, many of the observed usability issues reflect challenges that are likely to arise with similar platforms, emphasizing the importance of prioritizing user-centered design to address clinicians’ needs [16,17].

One of the platform’s key strengths lies in its intuitive and easy-to-navigate interface, particularly its scheduling and appointment booking functionality. Features such as appointment scheduling and automatic email reminders facilitate streamlined workflows and reduce the risk of missed sessions. However, the lack of integration with external calendars limits the platform’s broader utility, as therapists often manage their schedules across multiple systems. This aligns with previous findings highlighting how insufficient interoperability can hinder workflow efficiency in digital health tools [39]. Incorporating calendar interoperability could make digital platforms more adaptable to real-world practices, enhancing usability and reducing scheduling conflicts.

The patient profile creation feature demonstrated another usability strength, offering a comprehensive approach to data collection and ensuring quick and clear data entry. Nevertheless, certain limitations restrict its inclusivity and functionality. The binary gender options fail to accommodate diverse patient identities, and the inability to attach scanned identification documents complicates the process of working with certain patient groups, such as minors. These issues reflect a broader need for platforms to adopt inclusive design principles, such as customizable gender fields and support for uploading supporting documentation, to enhance usability and accessibility for diverse users [40].

Similarly, the system for handling informed consent forms reflects a mix of strengths and weaknesses. While the automatic sending of consent forms is valuable, the reliance on physical printing, signing, and scanning introduces inefficiencies, reducing the platform’s potential to streamline administrative tasks. Supporting digital signatures and enabling secure uploads of signed forms would align the platform with modern standards for digital efficiency, as observed in previous studies advocating for the use of technology to minimize manual processes [38].

The annotations and attachments section, which allows for flexible note-taking, underscores the importance of accommodating diverse practitioner preferences. While the unstructured format supports various documentation needs, requiring a laptop for note-taking can distract therapists during sessions and limit functionality for those who prefer using tablets or digital pens. Such device limitations reflect broader issues of compatibility that must be addressed to ensure platforms are adaptable to different clinical settings [39]. Adding offline functionality and synchronization features would further enhance accessibility, ensuring that therapists can work even in environments with limited connectivity.

The mandatory fields and detailed data sections illustrate the tension between standardization and flexibility. While mandatory fields ensure essential information is documented consistently, the frequent updating of rarely changing data, such as medical histories, creates inefficiencies. In addition, the overly medicalized structure of these fields may not align with therapeutic needs, particularly for less diagnostic-focused approaches. Simplifying data entry by introducing permanent fields for stable information and incremental updates could address these inefficiencies and improve alignment with diverse therapeutic approaches.

The dashboard for monitoring patient progress represents a key strength, offering an intuitive graphical display to visualize patient trajectories. However, the lack of dates on progress graphs limits therapists’ ability to contextualize changes over time, an important feature for treatment planning and trend analysis. Including time-stamped data would enhance the dashboard’s utility and align it with therapists’ needs for precision and longitudinal tracking [38,39].

The functionality for sending questionnaires highlights the platform’s potential to simplify data collection, but is constrained by its inability to include custom questionnaires. Allowing therapists to upload and manage custom questionnaires would increase adaptability to specific therapeutic needs. Similarly, the feature for generating and sending reports to patients, while efficient, lacks flexibility in controlling content and timing. Offering greater control over report customization and dissemination would align the platform’s functionalities with therapeutic goals and minimize potential patient confusion.

These findings underscore the need for usability improvements that extend beyond individual features to address how digital tools integrate into broader clinical workflows. The challenges identified reflect broader systemic issues noted in previous research, including the need for flexibility, inclusivity, and integration [39,40].

### On the Integration of Digital Platforms Into Psychotherapeutic Practices

The introduction of a structured digital system for documentation, such as Mindy, has raised significant tensions between the need for standardization and the inherent flexibility required in psychotherapeutic practice (first theme—“balancing standardization with the need for narrative and implicit information”). While structured fields and predefined scales facilitate consistency and quantitative analysis, they risk oversimplifying or omitting critical narrative details, such as tone, body language, and contextual observations. This issue aligns with previous literature, which suggests that EHRs in mental health care often interfere with the richness of documentation required for effective treatment [38]. The rigidity of standardized fields may constrain therapists’ ability to adapt documentation to individual patients, reducing the depth necessary to fully capture therapeutic dynamics [41]. This concern is particularly pronounced in psychoanalytic approaches, where patient narratives and nonverbal cues play a crucial role in treatment [40]. Addressing this challenge requires a balance between standardization for uniformity and flexibility to accommodate the personalized nature of psychotherapy. Looking forward, the emergence and popular use of artificial intelligence–based scribes [42] present an intriguing development that may reshape perceptions of platforms such as Mindy. AI scribes, which automatically transcribe and summarize clinical interactions, have the potential to reduce the documentation burden on clinicians while preserving the richness of narrative and implicit information. These technologies could complement platforms such as Mindy by enabling therapists to focus more on patient interaction rather than manual data entry. However, their widespread adoption raises new questions about data security, integration with existing systems, and the ethical implications of AI-mediated documentation.

The adoption of digital platforms presents both technical and operational challenges for psychotherapists, particularly regarding their integration into daily workflows (second theme—“challenges in embedding digital platforms into daily therapeutic workflows*”*). The study highlighted difficulties related to time management, computer literacy, and resistance to change. Experienced therapists, in particular, expressed reluctance to move away from established practices, perceiving digital tools as an additional workload rather than a resource for streamlining processes. Similar issues are noted in the literature, where the adaptation to EHRs has been shown to demand significant cognitive and operational adjustments [40]. Moreover, the study revealed concerns about how using digital tools, even in face-to-face sessions, can create a perceived barrier between therapist and patient, potentially disrupting the therapeutic alliance—a phenomenon also discussed in telehealth contexts [43]. In addition, concerns about data security were prevalent, with therapists emphasizing the importance of robust protections against unauthorized access, echoing findings by Golz et al [39]. To overcome these barriers, platforms such as Mindy must prioritize user-centered design, offering clear training and flexible integration strategies to minimize disruption to professional routines.

The final theme (“navigating the trade-offs between transparency and confidentiality in therapeutic data sharing”) revolves around the management of transparency and confidentiality in sharing sensitive therapeutic data. While transparency can strengthen the therapeutic alliance [44], unmediated sharing of session notes poses risks of misinterpretation and patient distress. For example, preliminary observations or speculative diagnoses might be misunderstood as definitive, leading to unnecessary anxiety or erosion of trust. This study supports previous literature advocating for selective and context-sensitive sharing of documentation [45]. In particular, therapeutic notes may be better suited for selective disclosure, depending on the stage of treatment and the specific needs of the patient. Digital platforms must, therefore, incorporate customizable sharing options to ensure communication is adapted to the therapeutic context while maintaining the foundation of trust essential to effective psychotherapy.

### Conclusions

This study examined the integration of digital technologies into psychotherapy [1,15,38], focusing on usability and broader implementation challenges associated with platforms such as Mindy. While EHRs and DMHPs offer benefits such as improved documentation, enhanced communication, and timely information retrieval [38], their adoption in psychotherapeutic practice is hindered by challenges related to standardization, workload, usability, and impacts on the therapeutic relationship [38]. These challenges are particularly relevant in the context of ROM, a practice that digital platforms are intended to support but that also introduces specific constraints [5].

The usability analysis of Mindy revealed that while its structured fields and predefined formats facilitate consistency and support ROM implementation, they limit therapists’ ability to personalize documentation. This lack of flexibility affects the capacity to capture the complexity and depth of therapeutic interactions, which is essential for tailoring treatments to individual patients. In addition, the inductive analysis identified 3 main dimensions that represent broader challenges of incorporating digital platforms into psychotherapeutic workflows.

Understanding how digital tools can be effectively integrated into clinical practice necessitates approaches that focus on the human-computer interaction, as emphasized by usability studies [17,26,27,27,28]. These studies, which observe how users interact with technology in real-world settings, provide valuable insights into the challenges faced by professionals. By capturing both immediate usability issues and broader themes emerging from these interactions, the study demonstrated how usability methods are critical not only for identifying technical barriers but also for uncovering contextual factors that influence the adoption of digital tools in psychotherapy.

In contributing to the broader goals of the OutProFeed project, this study highlighted the importance of a multidisciplinary approach to improving Mindy’s usability and integrating it into professional workflows. The findings underscore the need for collaboration between clinicians, software engineers, and researchers, each bringing distinct expertise and perspectives. While these groups often approach the design and implementation of digital tools with different priorities and terminologies, fostering a shared understanding is essential to ensure that technical features align with clinical needs.

## Supplementary material

10.2196/75885 Multimedia Appendix 1 Mindy’s interface.

## Abbreviations

APA: American Psychiatric Association
DMHP: digital mental health platform
EHR: electronic health record
FDA: Food and Drug Administration
GDPR: General Data Protection Regulation
ROM: routine outcome monitoring
