# Supplementary material for: Assessment of a Digital Platform for Routine Outcome Monitoring in Psychotherapy: Usability Study and Thematic Analysis
Source: JMIR Med Inform. 2025 Sep 30;13:e75885. doi: 10.2196/75885 (PMC12483338; doi:10.2196/75885)
Supplement: Multimedia Appendix 1 [file medinform-v13-e75885-s001.docx]

**Multimedia Appendix 1**

**Mindy’s interface**


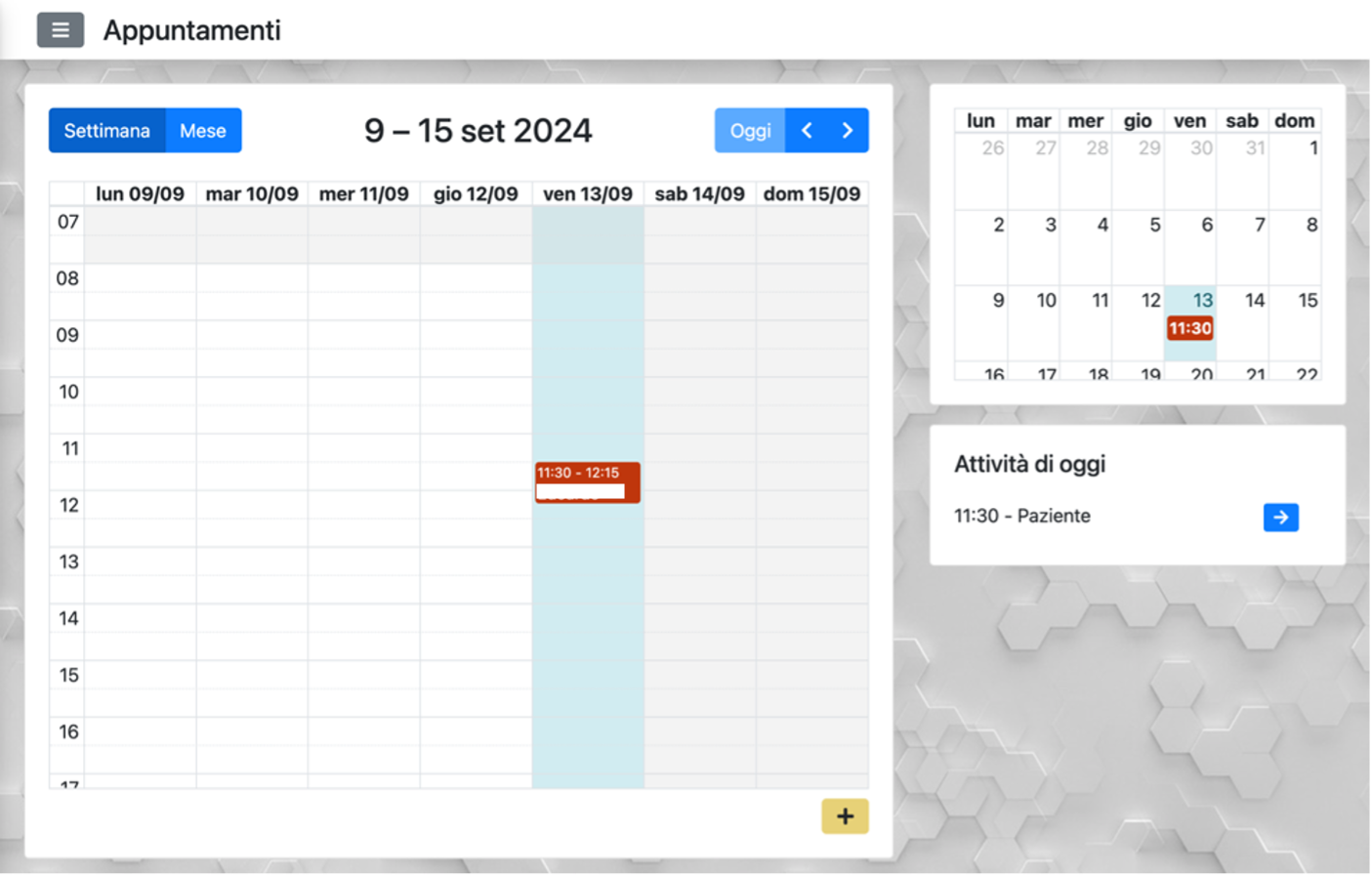


Agenda and appointment booking

Patient profile creation


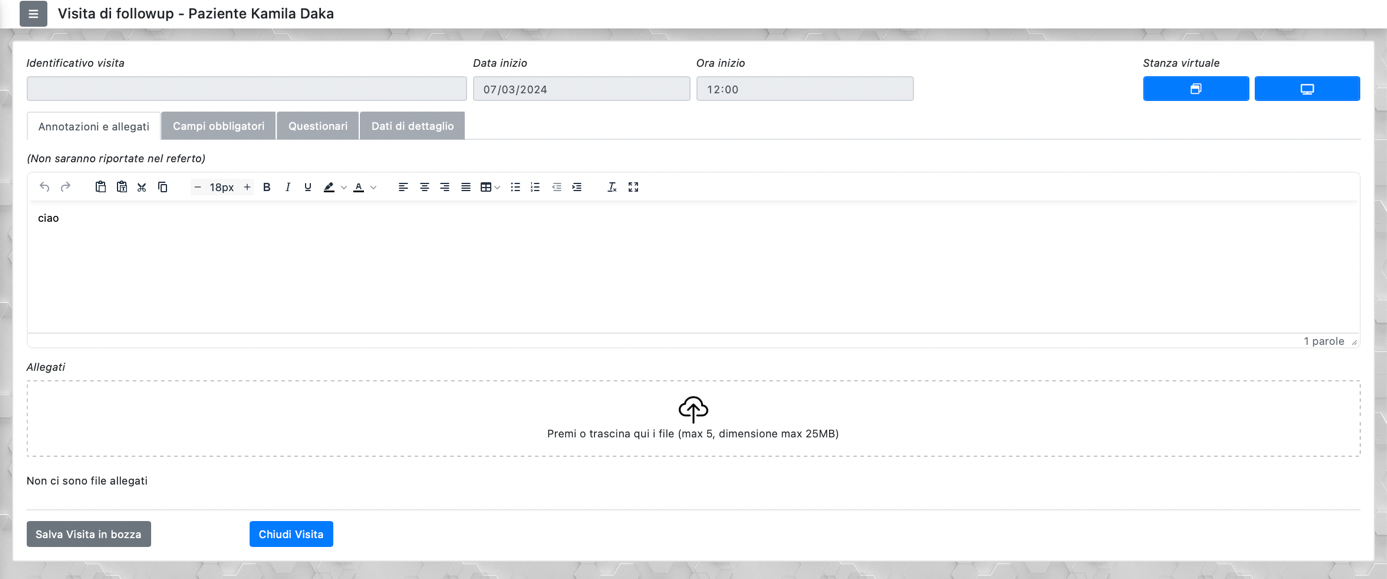


Management of Session Data with the Patient – “Annotations and Attack”


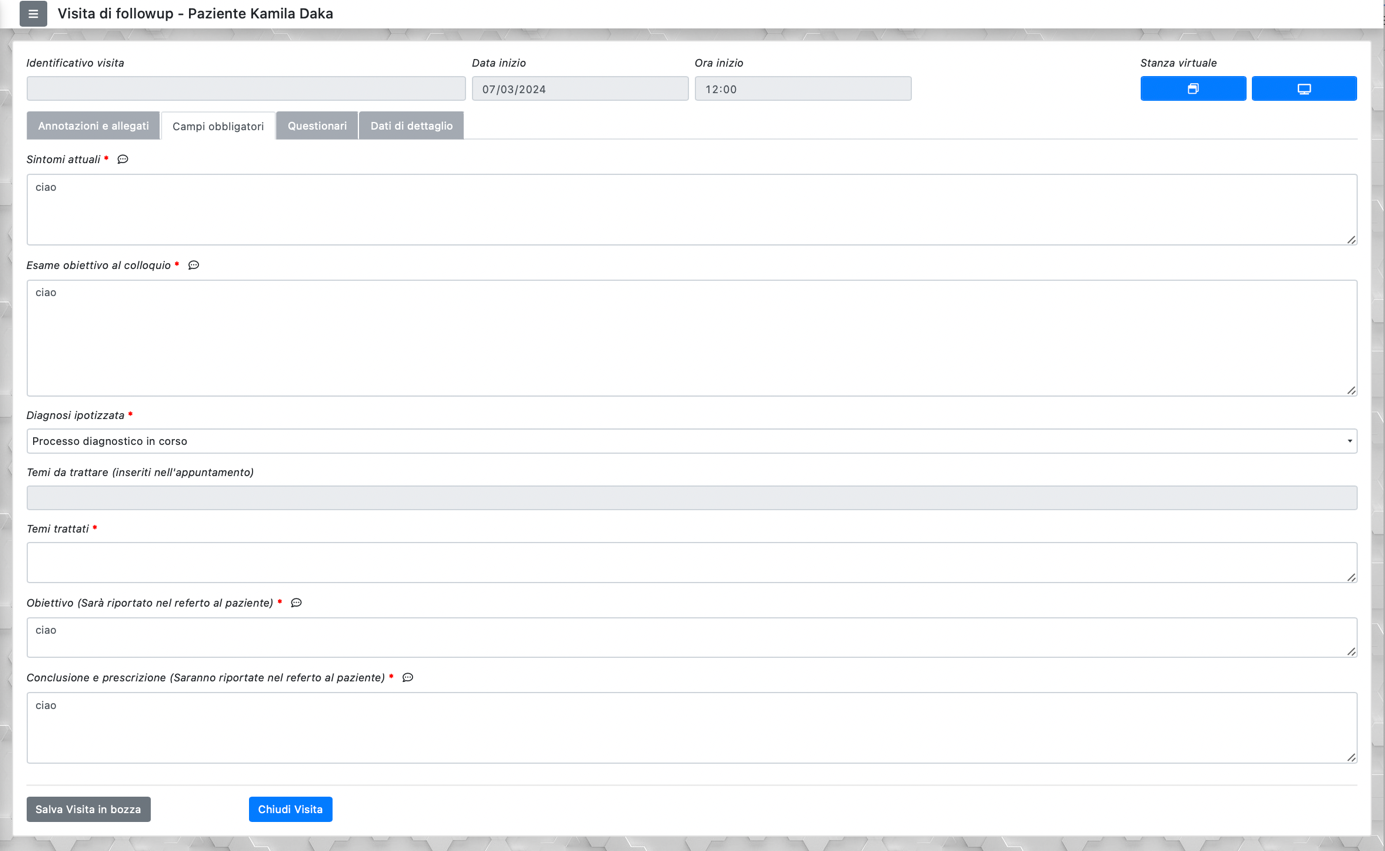


Management of Session Data with the Patient – “Mandatory Fields”


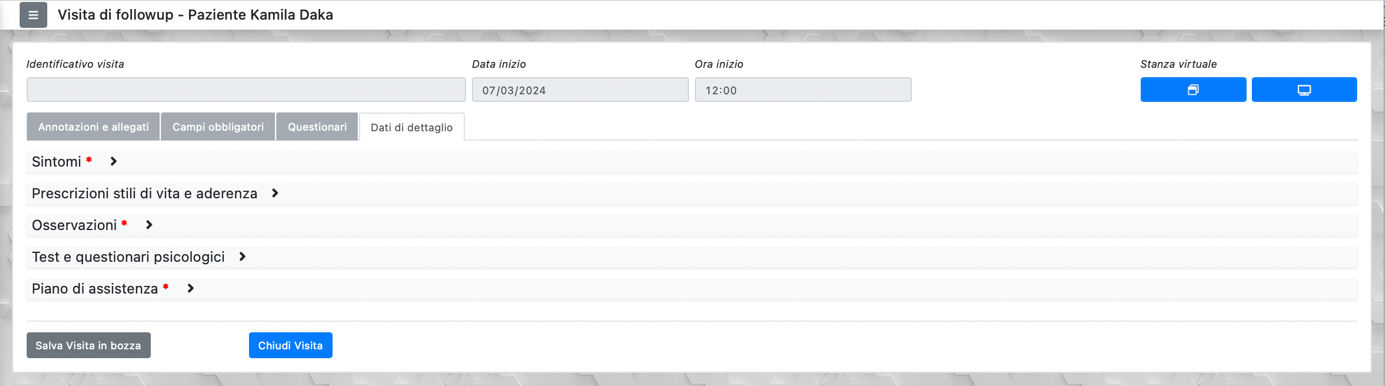


Management of Session Data with the Patient – “Detail Data”


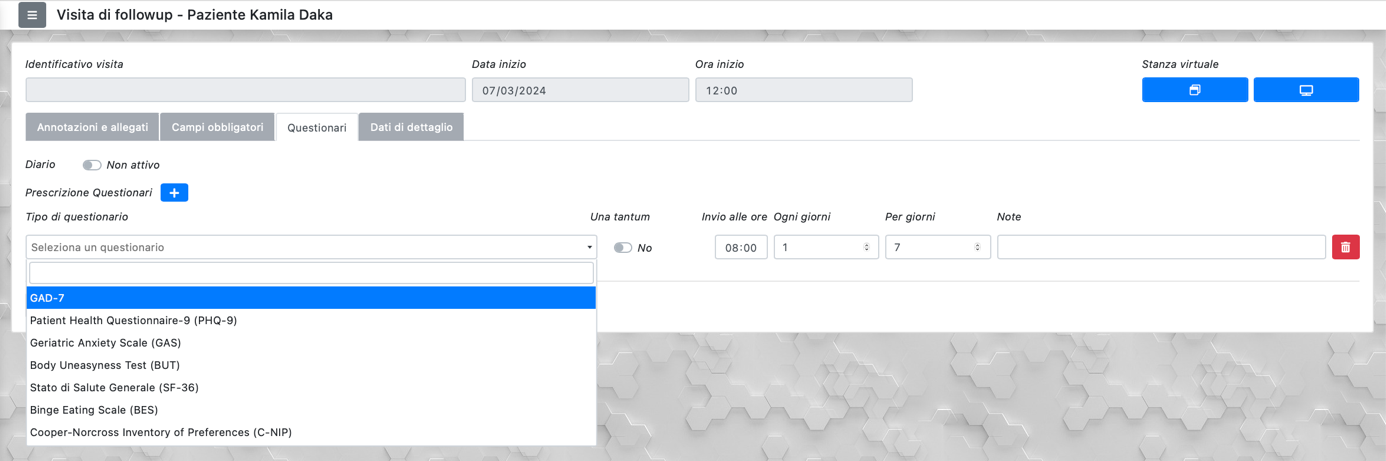


Management of session data with the patient - “Questionnaires”


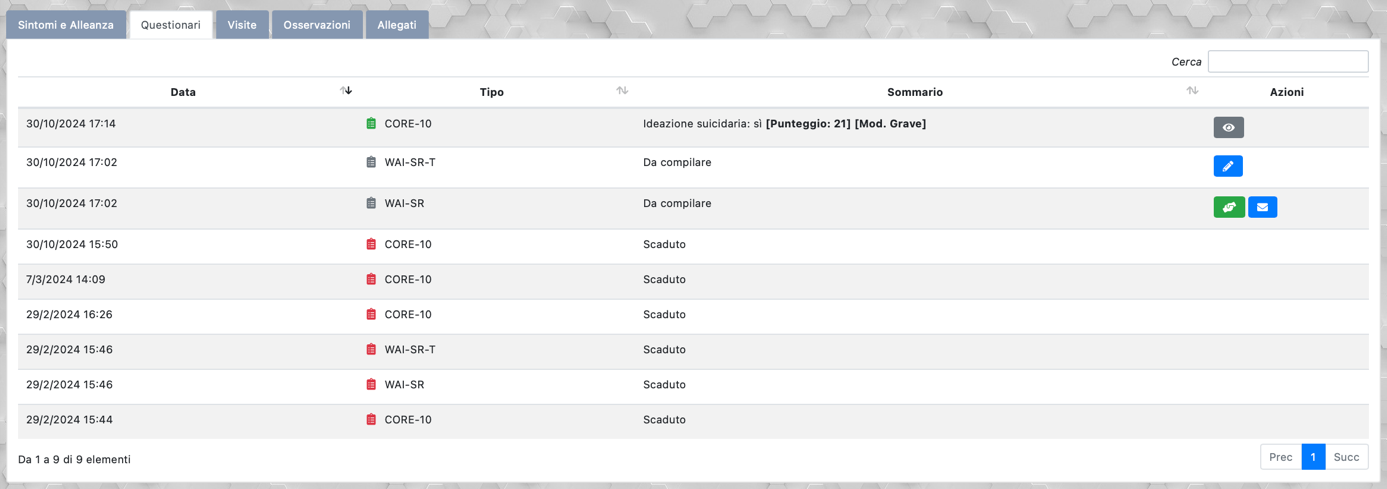


Management of session data with the patient - “Questionnaires”


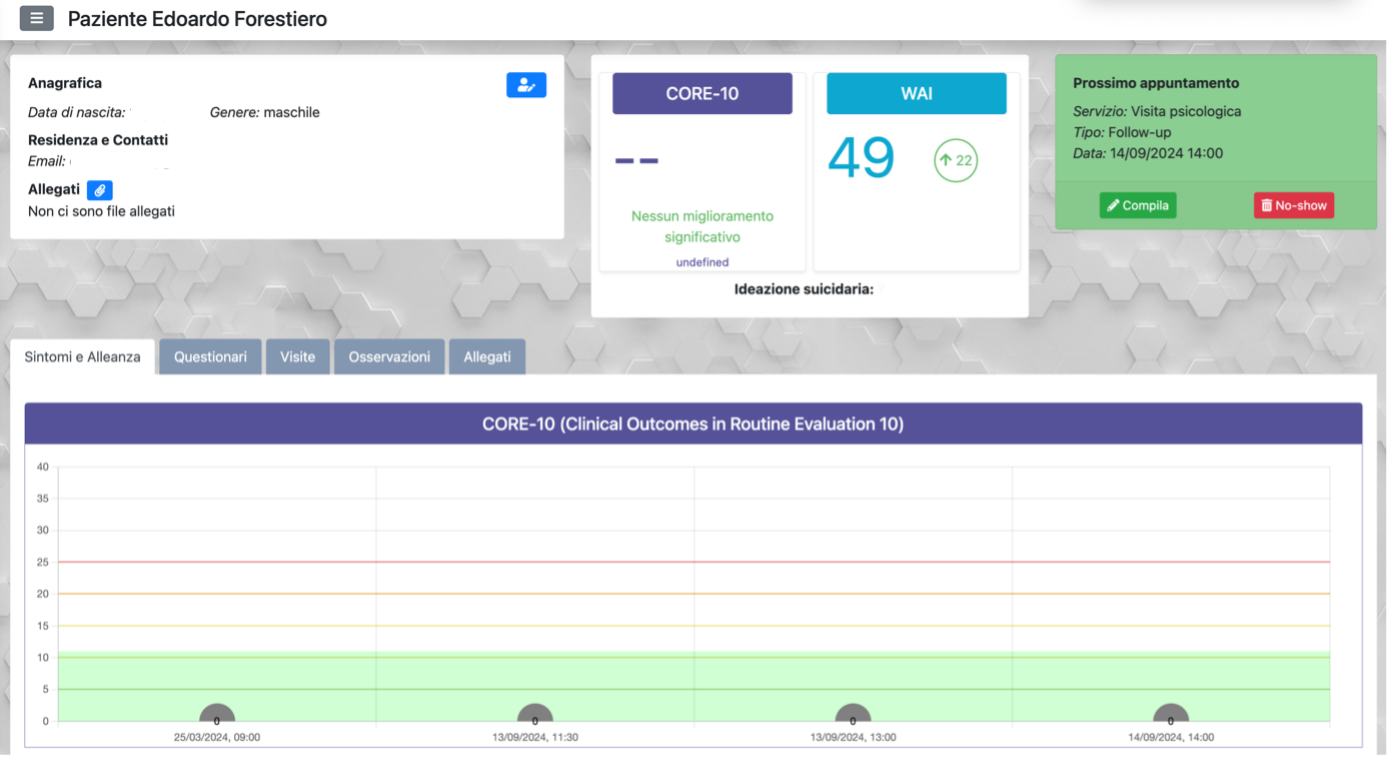


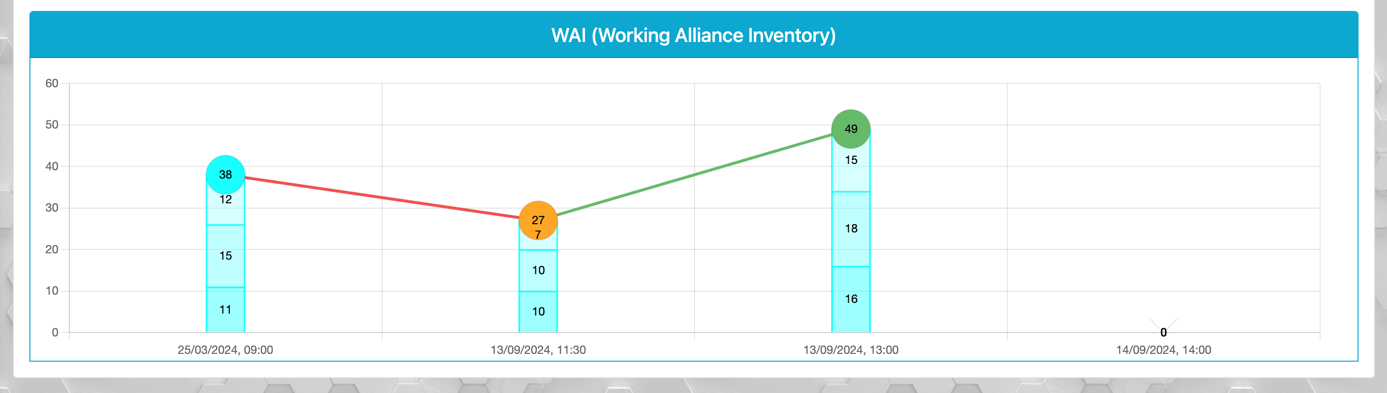


Dashboard to Monitor Patient Progress and the Therapeutic Alliance
